# Acquired hemophagocytic syndrome: comment to the case report

**DOI:** 10.4155/fso.15.29

**Published:** 2015-11-01

**Authors:** Chiara Ciccarese, Francesco Massari, Giampaolo Tortora

**Affiliations:** 1 Medical Oncology, Azienda Ospedaliera Universitaria Integrata (A.O.U.I.), University of Verona, P.le L.A Scuro 10, 37134, Verona, Italy

**Keywords:** chemotherapy, hemophagocytic lymphohistiocytosis syndrome, synovial sarcoma

Hemophagocytosis represents the peculiar pathological feature of activated macrophages engulfing hematopoietic cells as a result of an immune dysregulatory disorder. This finding is the hallmark of a rare hyperinflammatory disease known as hemophagocytic lymphohistiocytosis (HLH).

HLH is a severe condition characterized by an abnormal ineffective immune response caused by an uncontrolled activation and proliferation of macrophages, natural killer (NK) cells and T-helper lymphocytes, leading to excessive cytokine production, subsequent amplification of pathologic immune stimulation, increased phagocytic activity and tissue damage [1]. Typically, heat shock proteins (HSP) are associated with a peculiar combination of clinical and biochemical features, including prolonged high fever, pancytopenia, hepatosplenomegaly, hypertriglyceridemia, hyperferritinemia and hemophagocytosis in the bone marrow. If undiagnosed or not promptly treated, HLH almost inevitably results in death.

First described in 1939 by Scott and Robb-Smith [2] as a life-threatening disorder characterized by histiocytic proliferation with erytrophagocytosis in the lymphoreticular system, it was named ‘histiocytic medullary reticulosis’ (HMR) and ascribed among malignant histiocytosis. In the following years, HSP were considered a hereditary immune dysregulatory disorder, called ‘familial hemophagocytic reticulosis’ [3], until Risdall described a viral association with HLH, highlighting a possible acquired etiology and therefore referring to it as ‘reactive hemophagocytic syndrome’ [4]. Subsequently, several cases have been involved in the genesis of sporadic HLH, including infections, malignancies, autoimmune diseases and metabolic conditions.

Therefore, HLH can be etiologically classified into a primary (genetic) or a secondary (acquired) disorder. All forms of HLH share hereditary or acquired immune defects characterized by impaired function of NK cells and cytotoxic T cells.

Primary HLH includes two groups: first, the familial hemophagocytic lymphohistiocytosis syndromes (FHLH1–5), in which HLH is the sole manifestation of the disease, and second, immunodeficiency syndromes associated with albinism (Chédiak Higashi syndrome, Griscelli syndrome type II, Hermansky-Pudlak syndrome type II) or other primary immunodeficiencies (i.e., X-linked lymphoproliferative disorder type I or II), where the hemophagocytosis is a part of a complex landscape of clinical manifestations [5]. Although not yet entirely identified, the known genetic abnormalities cause defect in proteins (like perforin, MUNC13.4, syntaxin-11 and STXBP2) that play a key role in granule-dependent lymphocyte cytotoxicity, impairing the maturation or the secretion cytotoxic granules during target cell killing, thus prejudicing the immune response [6]. Hereditary HLH usually occurs in infants or young children, with clear familial inheritance, is associated with high risk of recurrence and short life expectancy without hematopoietic cell transplantation.

Secondary HLH, currently more common than primary forms, affects older children or adults without known genetic cause. Several pathological conditions may act as a trigger for the development of acquired HLH.

Infections, mainly viral and Epstein–Barr virus (EBV) associated, but also caused by bacterial, fungal and parasitic organisms, play a crucial role. However, infections may also represent the stimulus responsible for an acute exacerbation of primary forms of HLH; moreover, there is often an overlap between HLH secondary to infection and a septic process, whose differential diagnosis is extremely complex [7].

HLH is associated with several disorders of the immune system, including autoimmune disorders, being lupus erythematosus the most frequently reported one, acquired immune deficiencies (such as AIDS) or immune disorders after hematopoietic stem cell transplantation. Macrophage activation syndrome can be considered as a variant of HLH developing in autoinflammatory diseases, principally in systemic juvenile idiopathic arthritis. Interestingly, also various metabolic disorders, including lysinuric protein intolerance, multiple sulphatase deficiency and alterations of propionate metabolism are known to be potential triggers for HLH [8].

HLH is known to develop in patients with malignancies, mainly lymphoproliferative disorders. Often, infections promote HLH in the context of a compromised immune system due to chemotherapy or cytokines produced by the tumor cells or infected T lymphocytes [9]. Among hematological malignancies, lymphomas or leukemias of T or NK cell lines, which are frequently related to EBV infection, are the most frequently associated with HLH [5,10].

The association between hemophagocytosis and solid tumors is anecdotal and refers almost exclusively to HLH developed in patients with mediastinal germ cell tumors [11], pediatric neuroblastoma and rhabdomyosarcoma, hepatocellular carcinoma [12], metastatic melanoma [13], squamous cell carcinoma of the neck [14], lung cancer [15], renal cell carcinoma [16], prostate cancer [17] and colon cancer [18].

HLH is the result of uncontrolled hyperinflammatory reaction to various types of triggers. The main physiopathological mechanism is an abnormal activation and proliferation of macrophages and CD8^+^ lymphocytes, and prominent cytokines production (including IFN-γ, sIL-2R, IL-1, TNF-α, IL-6 and IL-18) induced by T-helper lymphocytes [19,20]. This hypercytokinemia leads to an autoamplification loop of macrophages and cytotoxic lymphocyte activation resulting in tissue damages. While the pathogenesis of primary HLH is largely understood, the mechanisms underlying acquired HLH are not yet fully recognized [21,22].

In genetic HLH, hereditary mutations affect the transport, release and/or activity of cytolytic granules in NK cells and cytotoxic T lymphocytes (CTL). Cytotoxic granules contain perforin and granzymes that induce apoptosis of target cells. Moreover perforin is important for the down-regulation of the immune response. As a result, despite NK and CTL granule-dependent cytotoxic activity is impaired, they can still activate and secrete cytokines, supporting a persistent activation of the immune response with tissue damage [22].

Different pathogenetic mechanisms can support acquired HLH. In most cases, there is not an impairment of lymphocyte cytotoxic activity, although some heterozygous mutations in genes that regulate the innate immune response and the NK function have been correlated with secondary HLH [23]. In case of infections, mainly by intracellular microorganism, the pathogenesis lies in a direct activation of Toll-like receptors with disproportionate activation of normal macrophage and T cells, leading to a detrimental Th1 cytotoxic response. The inability to deal with infections in immunocompromised patients can be a trigger for HLH development: cells of the innate immune response are continuously activated via pattern recognition receptors (such as Toll-like receptors) that respond to components of opportunistic bacteria, mycoplasma, fungi, and viruses, sustaining an uncontrolled immune response. In autoimmune disorders, non-antigen-specific triggering of innate immunity may represent the precipitating factor [21]. Several cytokines released by malignancies may induce HLH [5]. HLC can be an extremely rare consequence of the cytotoxic effects of chemotherapy, whose massive tumor cell destruction results in a pathological immune response, leading to excessive cytokine production [14,17,24].

Given the poor prognosis, timely diagnosis is crucial. There is no single feature that is pathognomonic for HLH, including hemophagocytosis. To facilitate the diagnosis, the HLH Study Group of the Histiocyte Society has established a panel of eight diagnostic criteria, five of which are required for HLH. The utility of these criteria is questionable because they lack specificity. Therefore, it is the coexistence of different clinical features (especially the triad of prolonged fever, hepatospleno- megaly- and cyto-penias) to become relevant [25].

Each clinical and laboratory finding is strictly related to the hyper-inflammation state of the disease. Macrophage and T-lymphocyte proliferation results in hepatosplenomegaly and peripheral lymphadenopathy. Fever is secondary to high concentrations of IL-2 and IL-1. Cytopenias is ascribable to TNF-α and IFN-γ overproduction that suppress hematopoiesis and direct hemophagocytosis. Coagulopathy is often reported, and due to macrophage secretion of plasminogen activators resulting in an accelerated conversion of plasminogen to plasmin and consequent hypofibrinogenemia. Disseminated intravascular coagulation can be a rare manifestation of elevated levels of TNF-α and IFN-γ. Serum ferritin elevation induced by IL-1β is highly sensitive and specific for HLH. Hypertriglyceridemia is the consequence of lipoprotein lipase inhibition by TNF-α.

As a severe condition, there should be no delay in starting therapy. The goal of treatment is to regulate the immune system dysfunction, suppressing the hyperinflammatory state. It is also important to treat the cause responsible for acquired HLH. Corticosteroids alone, or in association with intravenous immunoglobulin, represents the main therapy in patients with rheumatology-associated HLH (macrophage activation syndrome), but may be also active for others secondary forms of HLH. In the latter case, however, the lack of a quick response should result in the promptly set up of full therapy, consisting in immune-suppressive agents, cytotoxic drugs, biological response modifiers, and stem-cell transplantation as suggested by the HLH-94 protocol. Unfortunately, about 25% of patients fail to achieve complete remission with standard therapy. At the moment, there are no standard salvage therapies. No conclusive results are reported with the use of infliximab, daclizumab, alemtuzumab, anakinra and other agents [26].

Refractory patients generally have dismal prognosis, encouraging research aimed at a better understanding of disease pathogenesis so as to develop more effective therapies.

## References

[1] Janka GE (2007). Hemophagocytic syndromes. *Blood Rev.*.

[2] Scott RB, Robb-Smith AHT (1939). Histiocytic medullary reticulosis. *Lancet*.

[3] Farquhar JW, Macgregor AR, Richmond J (1958). Familial haemophagocytic reticulosis. *BMJ*.

[4] Risdall RJ, McKenna RW, Nesbit ME (1979). Virus-associated hemophagocytic syndrome: a benign histiocytic proliferation distinct from malignant histiocytosis. *Cancer*.

[5] George MR (2014). Hemophagocytic lymphohistiocytosis: review of etiologies and management. *J. Blood Med.*.

[6] de Saint Basile G, Ménasché G, Fischer A (2010). Molecular mechanisms of biogenesis and exocytosis of cytotoxic granules. *Nat. Rev. Immunol.*.

[7] Rouphael NG, Talati NJ, Vaughan C, Cunningham K, Moreira R, Gould C (2007). Infections associated with haemophagocytic syndrome. *Lancet Infect. Dis.*.

[8] Bode SF, Lehmberg K, Maul-Pavicic A (2012). Recent advances in the diagnosis and treatment of hemophagocytic lymphohistiocytosis. *Arthritis Res. Ther.*.

[9] Janka G, Imashuku S, Elinder G, Schneider M, Henter JI (1998). Infection- and malignancy-associated hemophagocytic syndromes. Secondary hemophagocytic lymphohistiocytosis. *Hematol. Oncol. Clin. North Am.*.

[10] Kojima H, Takei N, Mukai Y, Hasegawa Y, Suzukawa K, Nagata M (2003). Hemophagocytic syndrome as the primary clinical symptom of Hodgkin's disease. *Ann. Hematol.*.

[11] Sada E, Shiratsuchi M, Kiyasu J (2009). Primary mediastinal non-seminomatous germ cell tumor associated with hemophagocytic syndrome. *J. Clin. Exp. Hematop.*.

[12] Sakai T, Shiraki K, Deguchi M (2001). Hepatocellular carcinoma associated with hemophagocytic syndrome. *Hepatogastroenterology*.

[13] Cordel N, Le Corvaisier-Piéto C, Young P (2000). Hemophagocytic syndrome and metastatic melanoma: 3 cases. *Ann. Dermatol. Venereol.*.

[14] Aryal MR, Badal M, Giri S, Aryal S (2013). Haemophagocytic lymphohistiocytosis mimicking septic shock after the initiation of chemotherapy for squamous cell carcinoma of the neck. *BMJ Case Rep.*.

[15] Molad Y, Stark P, Prokocimer M, Joshua H, Pinkhas J, Sidi Y (1991). Hemophagocytosis by small cell lung carcinoma. *Am. J. Hematol.*.

[16] Chao CT, Kao CC, Lee SY (2012). Renal cell carcinoma with secondary hemophagocytic syndrome: a case report. *Can. Urol. Assoc. J.*.

[17] Declerck L, Adenis C, Amela E, Caty A, Adenis A, Penel N (2012). Prostate cancer related haemophagocytic syndrome: successful treatment with chemotherapy. *Acta Oncol.*.

[18] Yamada T, Ikeya T, Ogawa T (2002). A hemophagocytic syndrome-like condition after emergency colectomy for perforated colon cancer: report of a case. *Surg. Today*.

[19] Henter JI, Elinder G, Söder O, Hansson M, Andersson B, Andersson U (1991). Hypercytokinemia in familial hemophagocytic lymphohistiocytosis. *Blood*.

[20] Mazodier K, Marin V, Novick D (2005). Severe imbalance of IL-18/IL-18BP in patients with secondary hemophagocytic syndrome. *Blood*.

[21] Janka GE, Lehmberg K (2013). Hemophagocytic lymphohistiocytosis: pathogenesis and treatment. *Hematol. Am. Soc. Hematol. Educ. Program*.

[22] Ménasché G, Feldmann J, Fischer A, de Saint Basile G (2005). Primary hemophagocytic syndromes point to a direct link between lymphocyte cytotoxicity and homeostasis. *Immunol. Rev.*.

[23] Hazen MM, Woodward AL, Hofmann I (2008). Mutations of the hemophagocytic lymphohistiocytosis-associated gene *UNC13D* in a patient with systemic juvenile idiopathic arthritis. *Arthritis Rheum.*.

[24] Kounami S, Nakayama K, Yoshiyama M (2012). Early-onset hemophagocytic lymphohistiocytosis after the start of chemotherapy for advanced neuroblastoma. *Pediatr. Hematol. Oncol.*.

[25] Henter JI, Horne A, Aricó M (2007). HLH-2004: diagnostic and therapeutic guidelines for hemophagocytic lymphohistiocytosis. *Pediatr. Blood Cancer*.

[26] Jordan MB, Allen CE, Weitzman S, Filipovich AH, McClain KL (2011). How I treat hemophagocytic lymphohistiocytosis. *Blood*.

